# Numerical Investigations of Flow over Cambered Deflectors at *Re* = 1 × 10^5^: A Parametric Study

**DOI:** 10.3390/biomimetics10060385

**Published:** 2025-06-10

**Authors:** Gang Wang, Zhi Wang, Zhaoqi Jiao, Pihai Gong, Changtao Guan

**Affiliations:** 1 State Key Laboratory of Mariculture Biobreeding and Sustainable Goods, Yellow Sea Fisheries Research Institute, Chinese Academy of Fishery Sciences, Qingdao 266071, China; wanggang@ysfri.ac.cn (G.W.); gongph@ysfri.ac.cn (P.G.); guanct@ysfri.ac.cn (C.G.); 2 National Engineering Research Centre for Marine Aquaculture, Zhejiang Ocean University, Zhoushan 316022, China; wangzhi@zjou.edu.cn; 3 School of Navigation and Naval Architecture, Dalian Ocean University, Dalian 116023, China

**Keywords:** cambered deflectors, bionics, hydrodynamics, CFD, metamodeling

## Abstract

The cambered deflectors in aquacultural facilities are applied to enhance hydrodynamic efficiencies or enable flow fields to be fully developed. Given the anticipated improvements with the bio-inspired profiles or tandem configurations, the hydrodynamics of cambered deflectors with the above features are investigated at $Re=1\times 10^{5}$. The relationship between force coefficients and local flow behaviors for both bionic and non-bionic isolated deflectors, as well as tandem deflectors, is revealed using k−ω SST simulation. The dependencies of force coefficients on gap (*G*), stagger (*S*), and inclination angles (θ) in tandem deflectors are illustrated using an updated metamodeling workflow with simulated data. It is demonstrated that the variations of force coefficients over angles of attack are related to flow physics in boundary-layer regions. The non-bionic isolated deflector with the $\theta =10^{\circ }$ prevails as the decent performances of $C_{L}$ and γ globally, which is chosen in the following studies. Regarding tandem deflectors, θ plays a more vital role in drag coefficients ($C_{D}$) and lift coefficients ($C_{L}$), while the influence of *S* is not quite considerable compared to *G*. Aiming for cost minimizations and lift improvements, an optimized tandem case is obtained and justified with the superiorities in flow fields. This study has provided novel insights into the designs and optimizations of cambered deflectors in aquacultural engineering.

## 1. Introduction

**Figure 1:**
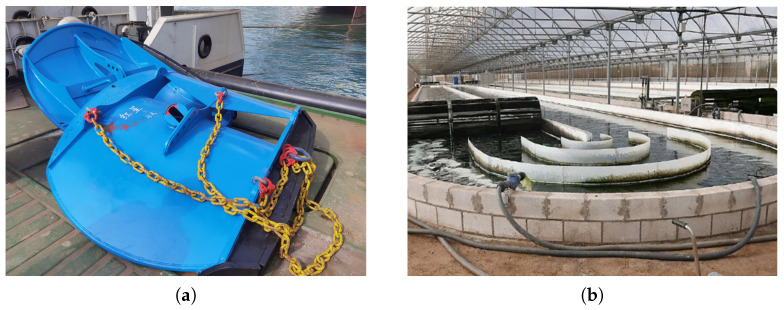
Applications of multiple deflectors in trawl doors and aquaculture tanks. (**a**) The prototype of a multi-deflector cambered trawl door; (**b**) The raceway pond for microalgae cultivation in a greenhouse [5].

One of the purposes for structural designs or optimizations of facilities in aquacultural engineering is to enhance hydrodynamic efficiencies, i.e., drag reductions and lift enhancements. It is paramount as the excessive drags lead to higher energy consumption and structural stresses, causing failures and accidents such as blade fractures of wind turbines [1]. Ineffective lifts or poor flow control can compromise the stabilities and performances of structures, such as inefficiency issues of monoplane-based trawl doors in fishing operations against the cambered designs [2]. Deflector structures, such as guide vanes, fins, or airfoil-shaped plates, have emerged as important passive flow control devices to address these needs by redirecting flow and altering pressure distributions. The most representative applications of deflectors in the fields of aquaculture are the multi-deflector trawl doors (Figure 1a) and raceway systems for algae cultivations (Figure 1b). A well-designed low-drag, high-lift trawl door can enhance fishing efficiency by keeping the net open horizontally while limiting the overall towing resistance, underscoring the value of lift-enhancing deflector designs in fishing operations [3]. Presently, trawl doors featuring multiple cambered boards or deflectors have been adopted extensively within the design or optimization phases. Likewise, guide vanes play a vital role in aquaculture tanks and raceway systems used for algae cultivation. In raceway ponds, recirculating flow often encounters “dead zones” and flow separation near bends, which increases energy dissipation and creates non-uniform residence times detrimental to algae growth [4]. To mitigate this problem, deflectors or guide vanes in the channel bends to straighten flow patterns and eliminate stagnant zones are introduced, indicating the significance of enhancing flow uniformity [4].

**Figure 2:**
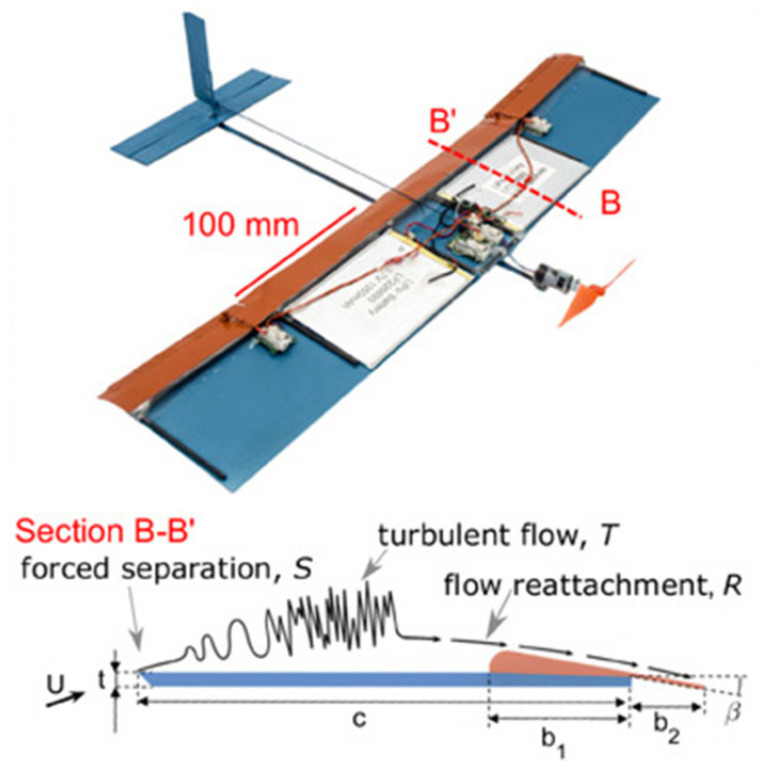
Application of the bioinspired Separated Flow airfoil in MAV [9].

The Reynolds numbers (Re) for practical operations of cambered deflectors in aquaculture are typically in the order of $10^{5}$, corresponding to high subcritical Re. The flow over thin-cambered plates or deflectors at subcritical Re is more complicated than that at the regime of critical Re due to non-negligible viscous effects [6]. Specifically, viscous effects are no longer confined to a negligible thin layer. Instead, laminar boundary layers tend to separate early on the airfoil and exhibit transitional phenomena that strongly affect performance [7]. In the range of high subcritical Re, Winslow et al. [2] noted that the laminar flow over the suction side of deflectors is subjected to a significant adverse pressure gradient close to the leading edge, resulting in the separation as the shear layer with low kinetic energies. Later, the separated shear layer would gain enough momentum from the freestream to reattach to the airfoil surface as a turbulent boundary layer, and the reattachment point is quite close to the trailing edge [8]. That hints at a more considerable separation bubble formed at the upper surface of the deflector, whereas the increase of Re can decrease the size of bubbles as a quicker reattachment is anticipated. Despite the non-linearities relationships between occurring loads and flow behaviors, it is commonly recognized that thin-cambered flat plates outperformed more conventional airfoils such as NACA0012 at these much lower Re [2,7]. The effects of thickness and camber of thin-cambered flat plates at the high subcritical Re are also discussed in detail. For further improvements in efficiencies, the overall shapes of airfoils made from thin plates are being innovated through bionic structures. Applied in micro air vehicles (MAV), a novel “Separated Flow” airfoil inspired by insect wings with sharp leading edges and corrugated surfaces is proposed by Di Luca et al. [9]. This design allows for sudden flow separation at the sharp leading edge, promoting a faster transition to turbulence. The rounded flap near the trailing edge also facilitates the reattachment of the separated layer as well (Figure 2). The improved lift performance of the bio-inspired separated flow airfoil has been supported by numerical simulations [10]. Nevertheless, the gaps in the hydrodynamics combining this bio-inspired structural upgrade with the cambered design are still preserved.

Previous studies have shown that arranging airfoils or deflectors in a tandem configuration can improve their aerodynamic efficiency, with the stagger distance, gap height, and decalage found to have particularly strong effects on the lift and drag characteristics [11,12], even at an ultra-low Re regime [13]. The relevance of force coefficients with gaps and staggers is considerably non-linear, but the reasonable configuration of tandem wings can increase lifts while delaying stalls [14]. For deeper insights, the flow interactions between airfoils or deflectors dominate the hydrodynamics variation. Originated from the dense collectives or schooling behaviors of eels, Kurt et al. [15] studied flow interactions of two low-aspect-ratio NACA0012 airfoils in an in-line and a staggered arrangement using particle image velocimetry (PIV) measurements. They have found that when the leader and follower interact in either arrangement, there are only minor alterations to the flow fields beyond the superposition of the flow fields produced by the isolated leader and follower [15]. However, the investigated Re is 4900, and the camber factor is not considered, rendering the applicability of our current research uncertain.

**Figure 3:**
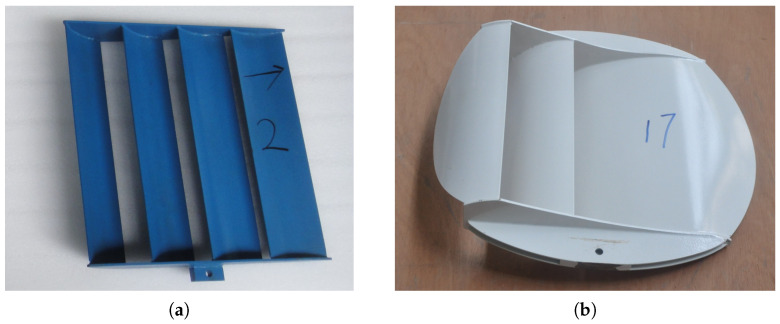
Overview of the multi-deflector cambered trawl doors. (**a**) Rectangular multi-wing cambered trawl door. (**b**) Oval-shape multi-wing cambered trawl door.

Here, special attention is paid to the cambered plates or deflectors applied in trawl doors, relating occurring forces with local flow behaviors and flow interactions. A more comprehensive grasp of flowing over cambered deflectors can provide novel insights into designs or optimizations of the current trawl doors. Retrospectively, a namely hyper-lift trawl door (HLTD) is invented with a cambered plate with double wing-end plates, and the leading edge is modified into an airfoil shape to reduce drags in Shen et al. [16]. Based on computational fluid dynamics (CFD) and physical measurements, You et al. [17] has improved the original conventional design by adding a steel shoe to prevent a decrease in the lift force with the low-aspect-ratio otter boards, taking the bottom and wing-tip flow effects [18] into consideration. From a more refined perspective, the feasibility of the shape of the primary cambered board of HLTD is further justified using the neural network method and genetic algorithms. The correlations between flow separation behaviors and local cambers are hence examined in You et al. [19]. A more innovative design by combining a pair of monoplane-type otter boards of the same size and shape is proposed in Takahashi et al. [3] and subsequently analyzed in You et al. [20], which is illuminated by the tandem airfoil concept to maximize the benefits of flow interactions. Herein the effects of gap-chord ratios, stagger angles, spanwise slits [21] or the assembly of flexible canvas [22] on force coefficients are discussed in a wide range of angles of attack. The force coefficients of a series of double-vane trawl doors are tested in Wang et al. [23], and they have found that the aspect ratio takes the most dominant position in force coefficients, then followed by gap ratios and camber ratios. In brief, the hydrodynamics implications of cambered boards applied in the structural optimizations of trawl doors to maximize the lift forces are addressed in the aforementioned works, but the pure gap-chord ratios, stagger angles, or gap ratios in the biplane-type trawl doors are still limited to exploit the hydrodynamic performance potential. It is further stated in Lin et al. [24] that the inclusion of the angle of attack for each board, which might cause the phase differences of downstream flow, can also compromise the hydrodynamic performances. Additionally, the applications of cambered deflectors to improve the hydrodynamic performances of trawl doors are pretty prevalent in forming multiple narrow slots, enforcing the fully developed flow conditions to reduce drags. The hydrodynamics of a rectangular multi-wing trawl door (see, e.g., Figure 3a) following this idea are studied numerically and experimentally [25,26]. The interrelations of thickness, camber ratios, aspect ratios, and installation angles of deflectors are included using metamodels, yet the spatial factors, such as gaps and staggers of deflectors, are neglected. Besides, the effects of sand clouds on the hydrodynamics of an oval-shape trawl door (see, e.g., Figure 3b) are also involved numerically [27], which is known to benefit from the desirable stability during handling over the irregular bathymetry in practice. The impacts of the multiple deflectors on local flow distributions are still nevertheless overlooked.

To the authors’ best knowledge, no existing research explores the relationship between the spatial factors and inclination angles of deflectors used in trawl doors simultaneously, and the flow interaction effects over the deflectors are not fully clarified yet. The design methodology for the spatial arrangement of cambered deflectors predominantly relies on empirically derived heuristics accumulated through decades of fishery engineering practices, but further optimization is expected to be driven by the accurate depictions of flow patterns around cambered deflectors. For this purpose, the flow past cambered deflectors at $Re=1\times 10^{5}$, including the isolated and tandem cases, is modeled using CFD simulations. The similarity criterion of Re is utilized to ensure the comparability of flow regimes between the prototype and the scale-down model. The investigated $Re=1\times 10^{5}$ is in accordance with the hydrodynamics of the full-scale trawl door with a towing speed from 0.5–3 kn for trawlers [26,28], as well as flow behaviors in aquaculture tanks. The profile of “Separated Flow airfoil” is applied for the isolated cambered deflector, and the hydrodynamic properties of both bionic and non-bionic ones are tested. The gap, stagger as well as the angle of attack are chosen as three control factors, which are found to have particularly considerable effects on hydrodynamic forces of tandem deflectors [11,13]. The metamodeling with CFD-based data is handled to illustrate their non-linear effects on force coefficients, and then the optimized tandem deflector satisfying the operational efficiency is finally obtained. It is of great significance that the flow patterns and interaction implications, including separations and reattachments within boundary-layer physics, are emphasized in this work, providing novel insights into the spatial optimization of tandem deflectors.

The structure of this paper is organized as follows: Section 2.1 introduces the investigated deflector models as well as the investigated parameters for arranging the tandem-configured deflectors. The numerical method, including the turbulence model, computational grids, solvers, schemes as well as the updated metamodeling workflow, are briefed in Section 2.2 and Section 2.3. Section 2.4 defines force coefficients, pressure, and skin-friction coefficients to capture the non-linear relationships between occurring loads and flow pattern evolutions. Based on the validation of the numerical framework (Section 3.1), the flow over the isolated deflectors with inclination angles, considering the bionic and non-bionic features, is studied in Section 3.2. Furthermore, the dependences of *G*, θ, and *S* on the force coefficients of and the underlying mechanism of flow interactions between tandem deflectors are discussed using the trained metamodel in Section 3.3, as well as the illustrations of the importance for each index. Finally, Section 3.4 showcases the superiority of the optimized tandem deflectors through the comparisons against the isolated deflector, presenting the alteration of flow distributions of the aft deflector through the interplaying effects.

## 2. Material and Methods

### 2.1. Deflector Models

**Figure 4:**
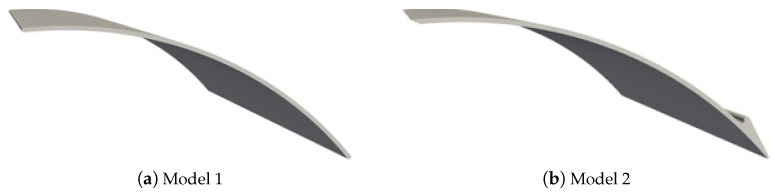
Overview of two investigated deflector models.

The first investigated deflector model (hereafter referred to as Model 1, Figure 4a) is derived from the mockup of a multi-wing cambered trawl door (Figure 3a). In line with the parametric studies conducted by Wang et al. [26], we have adopted an optimized relative camber r/c of 12% and an isotropic thickness of 1.53 mm for each deflector. Moreover, the topologies of the “Separated Flow” airfoil are applied for the second investigated deflector model (hereafter referred to as Model 2, Figure 4b). Here, the sharp leading edge and the rounded flap close to the trailing edge are applied for Model 2 while keeping the relative camber and the isotropic thickness unmodified as in Model 1.

**Figure 5:**
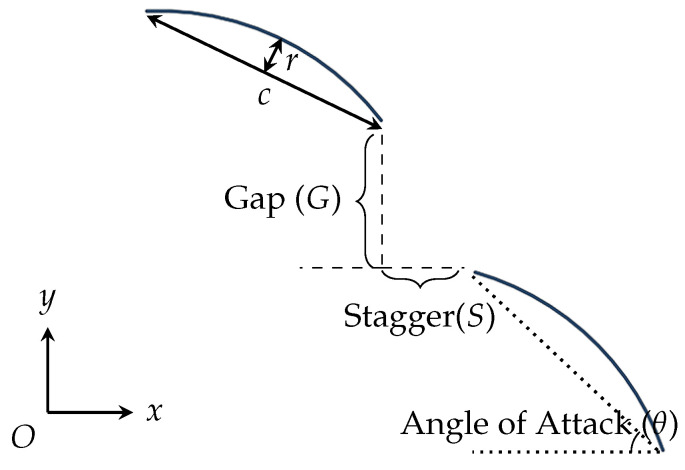
Definitions of the geometrical parameters in the tandem configurations of deflectors.

It should be noticed that the leader deflector and the follower are consistent in geometries but configured in a tandem pattern. The gap *G* and the stagger *S* are defined in Figure 5 for the tandem configuration, representing the vertical and horizontal spacings between the trailing edge of the fore deflector and the leading edge of the aft deflector, respectively. The angle of attack (θ) of the aft deflector is also chosen as the control parameters, while that of the fore deflector is determined through the hydrodynamics examination of the isolated case in Section 3.2. The position of the aft deflector can be configured within the downwash zone of the fore deflector through structural optimization, as demonstrated by Wang et al. [26]. While considering the limitations of wing endplates for trawl doors and engineering practices aimed at improving efficiencies, the investigated ranges for the parameters *G*, *S*, and θ are defined in Table 1. Special attention is needed that the upper deflector is placed forward to the lower one because of the marked improvements of lift forces compared to the other strategies [29]. The normalization of *G* and *S* with respect to *c* highlights the broad applicability of the hydrodynamic results in this study for both prototypes and scaled-down models.

### 2.2. Viscous Fluid Dynamics Solver

#### 2.2.1. Governing Equations

The mass and momentum of the incompressible viscous fluids in the whole domain are conserved through the two-dimensional continuity and unsteady Reynolds-Averaged Navier–Stokes (URANS) equations, which are given as

(1) $$\begin{array}{cc} \frac{\partial {\bar{u}}_{i}}{\partial x_{i}} & =0,\\ \frac{\partial {\bar{u}}_{i}}{\partial t}+{\bar{u}}_{j}\frac{\partial {\bar{u}}_{i}}{\partial x_{j}} & =-\frac{1}{\rho }\frac{\partial \bar{p}}{\partial x_{i}}+\frac{\partial }{\partial x_{j}}\left[\left(\nu +\nu_{t}\right)\left(\frac{\partial {\bar{u}}_{i}}{\partial x_{j}}+\frac{\partial {\bar{u}}_{j}}{\partial x_{i}}\right)\right]. \end{array}$$

where ${\bar{u}}_{i}$ with i=1,2 are the streamwise and spanwise mean velocity components. $\partial \bar{p}/\partial x_{i}$ and ρ denote the pressure gradient and the density of viscous fluids, respectively. ν is the fluid viscosity while $\nu_{t}$ is the turbulent eddy viscosity, and the latter can be deduced by the k−ω Shear Stress Transport (SST) model [30,31]. The standard k−ϵ model outperforms the high-Re flow modeling, whereas the intricate flow separations induced by the critical adverse pressure gradients in the near-wall region render more accurate modeling attempts futile. The k−ω SST model lies in the combination of Wilcox’s k−ω method [32] in the near-wall region and the standard k−ϵ model [33] accounting for freestream areas. It has been widely adopted in the numerical studies of two-dimensional flow past cylindrical structures [34], airfoils [35] as well as wall-mounted structures [36] at subcritical or high Re. The governing equations for the turbulent kinetic energy *k* and turbulent specific dissipation rate ω are given as

(2) $$\begin{array}{cc} \frac{\partial (\rho k)}{\partial t}+\frac{\partial (\rho u_{j}k)}{\partial x_{j}} & =\tilde{P}-\beta^{*}\rho \omega k+\frac{\partial }{\partial x_{j}}\left[\left(\nu +\sigma_{k}\nu_{t}\right)\frac{\partial k}{\partial x_{j}}\right],\\ \frac{\partial (\rho \omega )}{\partial t}+\frac{\partial (\rho u_{j}\omega )}{\partial x_{j}} & =\frac{\gamma }{\nu_{t}}\tilde{P}-\beta \rho \omega^{2}+\frac{\partial }{\partial x_{j}}\left[\left(\nu +\sigma_{\omega }\nu_{t}\right)\frac{\partial \omega }{\partial x_{j}}\right]+2\left(1-F_{1}\right)\frac{\rho \sigma_{\omega 2}}{\omega }\frac{\partial k}{\partial x_{j}}\frac{\partial \omega }{\partial x_{j}}. \end{array}$$

where $\tilde{P}$ the production term of *k* for the incompressible viscous fluids is approximated as

(3) $$\begin{array}{c} \tilde{P}=\min\left(\nu_{t}S^{2},10\beta^{*}\rho k\omega \right). \end{array}$$

where *S* is the strain rate. According to the k−ω SST model, $F_{1}$ plays in a vital role in blending the constant $\Phi_{1}$ from the Wilcox’s k−ω model [32] and the constant $\Phi_{2}$ from the standard k−ϵ model [33], then the constant Φ in the ultimate stage reads as

(4) $$\begin{array}{c} \Phi =F_{1}\Phi_{1}+\left(1-F_{1}\right)\Phi_{2}. \end{array}$$

with

(5) $$\begin{array}{cc} F_{1} & =\tanh(arg_{1}^{4}),\\ arg_{1} & =\min\left(\max\left(\frac{\sqrt{k}}{\beta^{*}\omega d_{w}},\frac{500\nu }{d_{w}^{2}\omega }\right),\frac{4\rho \sigma_{\omega 2}k}{CD_{k\omega }d_{w}^{2}}\right).\\ CD_{k\omega } & =\max\left(2\rho \sigma_{\omega 2}\frac{\nabla k\nabla \omega }{\omega },10^{-10}\right). \end{array}$$

where $d_{w}$ represents the distance to the nearest wall. The constants for $\Phi_{1}$ from the Wilcox’s k−ω model [32] are listed as

(6) $$\begin{array}{cc} \sigma_{k1}=0.5,\sigma_{\omega 1} & =0.5,\beta_{1}=0.0750,\\ \beta^{*}=0.09,\kappa =0.41,\gamma_{1} & =\beta_{1}/\beta^{*}-\sigma_{\omega 1}\kappa^{2}/\sqrt{\beta^{*}}. \end{array}$$

and the constants for $\Phi_{2}$ from the standard k−ϵ model [33] are given as

(7) $$\begin{array}{cc} \sigma_{k2}=1.0,\sigma_{\omega 2} & =0.856,\beta_{2}=0.0828,\\ \beta^{*}=0.09,\kappa =0.41,\gamma_{2} & =\beta_{2}/\beta^{*}-\sigma_{\omega 2}\kappa^{2}/\sqrt{\beta^{*}}. \end{array}$$

Finally, the $\nu_{t}$ in Equation (1) with the upgraded version of the k−ω SST model [31] is derived as

(8) $$\begin{array}{cc} \nu_{t} & =\frac{\rho a_{1}k}{\max(a_{1}\omega ,F_{2}S)}. \end{array}$$

with

(9) $$\begin{array}{cc} a_{1} & =0.5532,\\ F_{2} & =\tanh(arg_{2}^{2}),\\ arg_{2} & =\max\left(\frac{2\sqrt{k}}{\beta^{*}\omega d_{w}},\frac{500\nu }{d_{w}^{2}\omega }\right). \end{array}$$

#### 2.2.2. Computational Domain, Grids and Boundary Conditions

**Figure 6:**
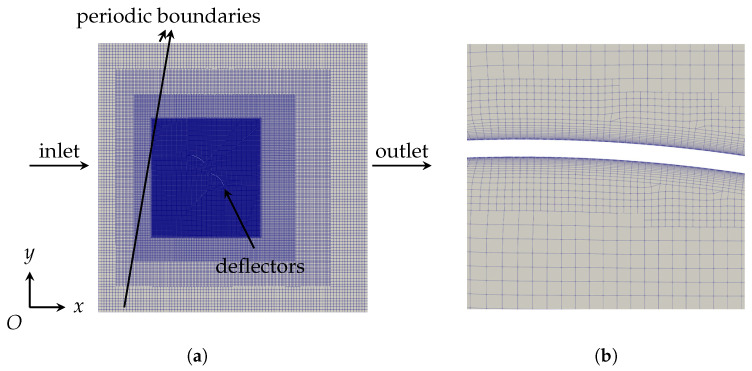
Computational domain and grids. (**a**) schematic figure; (**b**) close-up of the near-wall grids with $y^{+}<1$.

As depicted in Figure 6, the geometric dimension of the two-dimensional computational domain is determined as 20c×20c, while the tandem deflectors are placed at 1/3 of the length and the middle in the width and height directions. The purpose of the configuration of tandem deflectors is to ensure the fully developed flow over structures, and Yin et al. [13] has confirmed the trivial effects from side boundaries as well. The $U_{0}$ and the isotropic turbulence parameters are employed at the inlet boundary. *k* and ω are initialized as

(10) $$\begin{array}{cc} k & =\frac{3}{2}{\left(0.16Re_{d_{h}}^{-1/8}U_{0}\right)}^{2},\\ \omega & =\frac{k^{1/2}}{(\beta^{*}{)}^{1/4}L}. \end{array}$$

with $Re_{d_{h}}^{-1/8}$ the Reynolds number based on the pipe hydraulic diameter $d_{h}$, and *L* give the turbulence length scale as calculated in Brørs B [37]. The Neumann boundary is applied at the outlet while leaving the pressure equaling zero. The lateral boundaries are specified as the periodic boundary conditions, which simulate an infinite domain by allowing disturbances to wrap around seamlessly, thereby eliminating artificial edge effects. Furthermore, the non-slip condition is employed for the deflector model.

The computational grids are generated using the blockMesh and SnappyHexMesh utilities. In this process, staggered grids are first castellated and then snapped with deflectors, ensuring the quality of the grids is maintained. The computational domain is initialized with a uniform 95 × 95 mesh via blockMesh, followed by four levels of nested refinement in the inner regions. Each refinement level systematically reduces the grid size to 50% of its parent layer across *x*- and *y*-axes, achieving a geometric progression ratio of 1:2 across successive levels. Moreover, a total number of 20 layers in the proximity of the near-field region is generated with the expansion rate 1.18 (Figure 6b), satisfying that the first layer is inside the laminar sub-layer of the boundary-layer region, i.e., $y^{+}<1$. This arrangement allows for the effective capture of laminar flow and the transition to turbulence, which is relevant for understanding flow separation. Relying on the grid independence study in Section 3.1, the cell number of each case fluctuates around 450 k.

#### 2.2.3. Numerical Schemes

All simulations were carried out using the open-source CFD platform OpenFOAM (v2312). The transient solver pisoFoam, which employs a Pressure Implicit with Splitting of Operators (PISO)-based algorithm to handle pressure–velocity coupling, was used to integrate the Navier–Stokes equations. To achieve a balance between numerical accuracy and stability, the convective terms were discretized with a second-order linear upwind stabilized transport (LUST) scheme that introduces minimal numerical dissipation. Diffusive terms were approximated using a second-order central differencing scheme, and temporal integration was performed with a second-order backward differencing scheme. An adaptive time-step Δt was employed for the unsteady simulations, adjusted dynamically to ensure that the Courant–Friedrichs–Lewy (CFL) number remained below 1.0. The pressure equation was solved using a generalized geometric–algebraic multigrid (GAMG) method, whereas the momentum equations were advanced with a Gauss–Seidel iterative solver augmented by additional smoothing to promote convergence.

### 2.3. An Improved Metamodeling Workflow with CFD Simulations

**Figure 7:**
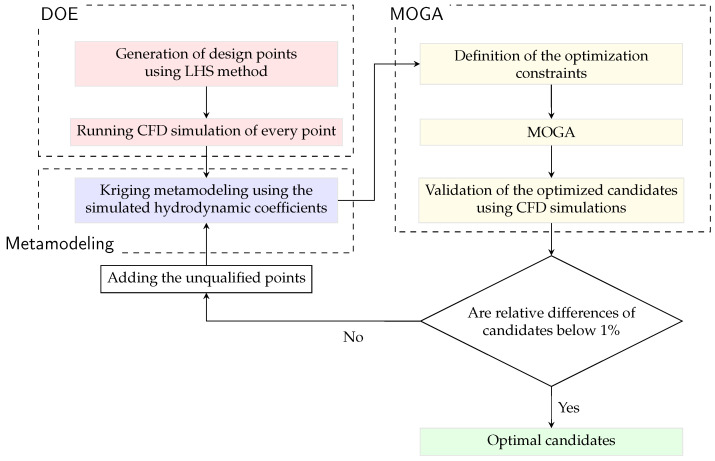
Flowchart of the metamodeling workflow with CFD simulations.

With the utilization of CFD data, Huang et al. [38] has proposed an integrated framework. It includes the design of experiments (DOE) and metamodeling to illustrate the non-linear correlations among response variables. The multi-objective genetic algorithm (MOGA) filters out the optimized candidates satisfying the predefined objectives. This unidirectional workflow compromises the predicted accuracies, but instead, the efficiency of the metamodel is emphasized as only a limited number of CFD cases need to be simulated. As shown in Figure 7, a refinement procedure to improve the metamodeling is straightforward by including the optimized candidates in the first round until the relative discrepancies of dependent variables of optimized candidates between metamodeling predictions and CFD data fall below 1% [26]. The robustness of this feedback is still weak since it focuses on the local refinement region. Thereby, the requirement of more points to meet the criterion in the context of intricate non-linearity correlations among variables is possible. To address this issue, the current workflow is updated by replacing the Central Composite Design (CCD) with the Latin Hypercube Sampling (LHS) method to generate design points in the inaugural sampling period. The details regarding three primary fractions of the workflow are introduced as follows.

- DOE: As opposed to the typical Monte Carlo random sampling, LHS is introduced in a fully stratified manner, which significantly improves the coverage of multi-dimensional design space [39]. Given an *m*-dimensional input space (with variables $X_{1},\ldots ,X_{m}$) and a desired sample size *N*, LHS partitions the range of each variable $X_{i}$ into *N* disjoint equiprobable intervals with equal probability. One random sample is then drawn from each interval of each $X_{i}$, yielding *N* values for each variable. Next, the *N* values obtained for each $X_{i}$ are randomly permuted and combined across variables to form *N* distinct *m*-dimensional sampling vector. Each such vector contains exactly one value from each $X_{i}$, and this construction ensures that each interval of every variable is represented exactly once across the entire sample set. In contrast, the exponential growth of the number of design points with respect to multi-dimensional cases is expected by classical designs like CCD strategy, which proves to be inefficient in engineering fields occasionally [40].
- Metamodeling: Kriging method gives the better unbiased predictions than the polynomial regression analysis [41], showing its flexibility to identify non-linearities with a limited number of observed data. The governing equation for the targeted response Γ(x) is formulated using two terms (Equation (11)). One is the mean response f(x) expressed by the polynomial basis, and the other refers to local responses L(x), which obeys the Gaussian distribution with zero mean and non-zero covariance. For the derivation of the unknown L(x) of interest, readers are referred to Wang et al. [26].
  (11) $$\begin{array}{cc} \Gamma (x) & =f(x)+L(x). \end{array}$$
- MOGA: Following the ideas of the regulated elitism concepts, MOGA is primarily characterized by the Non-dominated Sorted Genetic Algorithm-II (NSGA-II) [42], which aims to identify optimized solutions within multiple predefined constraints. The offspring are generated from the selected chromosomes based on the objectives, utilizing crossover and mutation processes inherent in MOGA. This approach balances the stability and randomness of the population. For a more detailed explanation of the procedures, readers can refer to Wang et al. [26]. In this study, the initial population consists of 3000 samples, with 600 samples generated per iteration. The crossover rate is set at 0.98, while the mutation rate is 0.01. To ensure the algorithm ultimately converges, the maximum allowable number of iterations is 20.

### 2.4. Data Statistics

The Reynolds number Re in this study is defined using the chord length *c* of a single deflector as the characteristic length scale, expressed through the conventional formulation as

(12) $$\begin{array}{c} Re=\frac{U_{0}c}{\nu }. \end{array}$$

where $U_{0}$ is the freestream velocity and ν is the kinematic viscosity. Focusing on lift enhancements and drag minimizations of cambered deflectors or trawl doors, the mean drag coefficient $C_{D}$ and lift coefficient $C_{L}$ are utilized for analyzing the mean drags $F_{D}$ and lifts $F_{L}$ on tandem deflectors with the whole exposed area *A*. The lift-drag ratio γ is also utilized to imply the hydrodynamics efficiencies of structures. They are written as follows:

(13) $$\begin{array}{cc} C_{D} & =\frac{2F_{D}}{\rho AU_{0}^{2}},\\ C_{L} & =\frac{2F_{L}}{\rho AU_{0}^{2}},\\ \gamma & =\frac{C_{L}}{C_{D}}. \end{array}$$

A better understanding of the hydrodynamics of deflectors is to reveal the flow separation physics over the suction side of the plate. The skin-friction coefficient $C_{f}$ and the pressure coefficient $C_{p}$ are thus utilized in this study. It is accounted for the evaluation of the separation position or the potential reattachment of turbulent boundary layers, which is relevant to the evolution of laminar separation bubbles (LSB). The formulae for $C_{f}$ and $C_{p}$ are calculated as follows

(14) $$\begin{array}{cc} C_{f} & =\frac{2|\tau_{w}|}{\rho U_{0}^{2}},\\ C_{p} & =\frac{2\left(p-p_{\infty }\right)}{\rho U_{0}^{2}}. \end{array}$$

where $|\tau_{w}|$ is the modulus of the local wall shear stress, *p* is the local static pressure while $p_{\infty }$ is the far-field pressure without any disturbance. In a similar manner, the aforementioned parameters are commonly adopted to describe the aero-efficiencies of airfoils or thin flat plates [2], given that there are relevant correlations between resultant forces and local flow separations.

## 3. Results and Discussions

### 3.1. Validations of the Numerical Method

**Figure 8:**
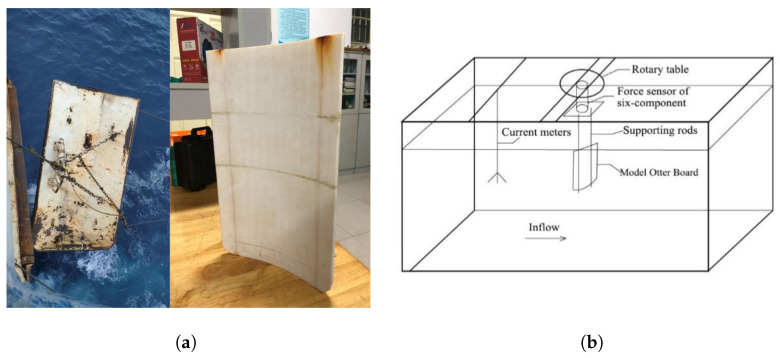
Schematic of the validated trawl door and the flume tank [43]. (**a**) Prototype and model of the cambered trawl door. (**b**) Placement of the trawl door model in the flume tank.

A validation study of the numerical method is performed using the experimental case [43] of a cambered trawl door model, with allowance for the geometrical similarities (Figure 8a). As illustrated in Figure 8b, the trawl door model is fixed with a supporting rod in the middle of a 4.0 m (length) × 1.2 m (width) × 1.0 m (water depth) flume tank, and the drag and lift forces of the cambered board in the steady freestream are measured using a six-component force sensor. The flow field around the validated structure is assumed to be in the high subcritical Re regime, preserving the similarity of turbulence fluctuations for the physical test and numerical simulations. In this section, a series of cases regarding inclination angles from $10^{\circ }$ to $40^{\circ }$ are compared experimentally and numerically. Herein, the independence test of computational grids is also carried out to eliminate the unpredicted numerical dissipation by grids. It is described in Table 2 that the $C_{L}$ is converging while $C_{D}$ experiences minor oscillations, with the refinement of outer grid size. The relative divergences (RD) of the numerical results against the measured data fall below 3% as long as the outer grid size reaches 0.02, which is satisfied and adopted in the following studies.

Over four cases depending on θ, the comparisons of the simulated $C_{D}$ and $C_{L}$ against the experimental and simulated results in Li et al. [43] are presented in Table 3. It is considerably opposed to the present study that the standard k−ϵ model, along with the explicit algebraic Reynolds stress model (EARSM), accounts for turbulence modelings, and the unstructured tetrahedral grids are snapped with the structure for the numerical treatment of Li et al. [43]. The maximum RD in the case of $\theta =40^{\circ }$ through the previous methodology is 14.43%, while that in the present study shows 5.17%. The numerical method, including the k−ω SST model in this paper, has been substantiated. In addition, the rising trend of RD of $C_{L}$ between the previous modeled results and the experimental data can be observed with the larger θ. A possible explanation lies in the ineffective ability of the k−ϵ EARSM model to capture the potential flow separations close to near-wall vicinities, which might lead to the spurious predictions of pressure fields at both sides of the otter board and inaccurate $C_{L}$.

### 3.2. Phase 1: Flow over an Isolated Deflector with the Variation of Inclination Angles

#### 3.2.1. Force Coefficients

**Figure 9:**
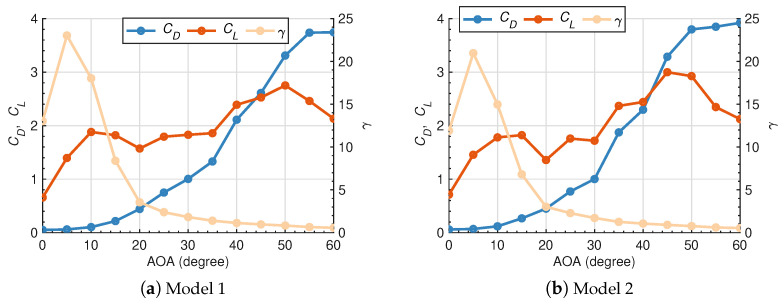
Force coefficients of two kinds of isolated deflectors with the variation of inclination angles.

In the inaugural stage, the hydrodynamics of two kinds of isolated deflectors with respect to inclination angles are analyzed and revealed, aiming to locate the optimized case for maximizing lift performances and efficiencies. Figure 9 shows $C_{D}$, $C_{L}$ and γ for both cases of $\theta =0^{\circ }$–$60^{\circ }$ with the forwarding step of $5^{\circ }$. With regards to the common rules observed in both models, $C_{L}$ increase abruptly, but $C_{D}$ rises slightly at $\theta =0^{\circ }$–$10^{\circ }$, resulting in the global peak of γ=23.03 and γ=20.96 at $\theta =5^{\circ }$, respectively. Then, it is of great interest that $C_{L}$ experiences perturbation or even decline during $\theta =10^{\circ }$–$30^{\circ }$, whereas $C_{D}$ escalates and γ still drops steadily. $C_{L}$ has recovered to rise after $\theta =35^{\circ }$ until the ultimate peak at $\theta =50^{\circ }$ for Model 1 as well as $\theta =45^{\circ }$ for Model 2, respectively. It can be noticed that the unique variation patterns of $C_{L}$ are not identified in the previous works studying the hydrodynamics of cambered otter boards [43,44]. The discrepancies might arise from the impacts of wing-end plates, cambered or thickness factors in the above research, which are contrasted to the studied isolated deflector. However, the embedded boundary-layer physics deserves further studies to be revealed later.

It can be naturally concluded that the presence of sharp leading edges and rounded back flaps in Model 2 are of minor significance for the overall varying trend of $C_{D}$ and $C_{L}$ with θ. For Model 1, $C_{L}$ decreases rapidly while $C_{D}$ still increases over 3.00 when $\theta \geq 50^{\circ }$. This trend has indicated the onset of stall, which is generally related to the massive streamlined separation at the suction part of the deflector. For Model 2, the bio-inspired features have contributed to the overwhelming performance of lift forces over Model 1, especially for $\theta =30^{\circ }$–$60^{\circ }$. The maxima reaches approximately 3.00 while the former reaches 2.75 at the critical θ. However, the $C_{L}$ and γ for the Model 1 are moderately higher than the Model 2 for $\theta =0^{\circ }$–$30^{\circ }$. The maximum relative differences of $C_{L}$ and γ are separately 15.80% and 23.22%. It is contradicted by the published numerical results [10], and the potential reasons lie in the introduction of the camber factor, which behaves more prevailed at the subcritical Re regime [2]. The Re investigated in Zhang et al. [10] is an order of magnitude smaller than that in the current research, which could bring about the discrepancy.

#### 3.2.2. Characteristics of Flow Fields

**Figure 10:**
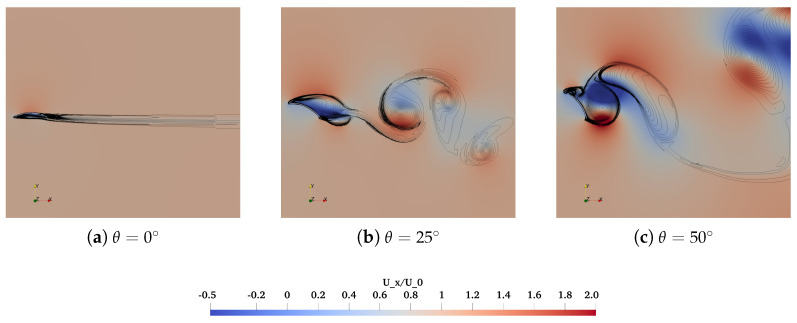
Instantaneous non-dimensional streamwise velocity $U_{x}/U_{0}$ and spanwise vorticity $c\omega_{z}/{U_{0}}^{2}$ fields around Model 1 with the variation of inclination angles. The solid lines donate the contours of $c\omega_{z}/{U_{0}}^{2}$ ranging from −20 to 20.

Flow patterns over the isolated deflector are subsequently characterized by the instantaneous non-dimensional streamwise velocity $U_{x}/U_{0}$ and spanwise vorticity $c\omega_{z}/{U_{0}}^{2}$ fields, as illustrated in Figure 10. On account of the marginal differences of force coefficients between two models for $\theta =0^{\circ }$–$40^{\circ }$, flow fields over Model 1 are principally concentrated other than the comparisons for $\theta =40^{\circ }$–$60^{\circ }$ at the last.

For $\theta =0^{\circ }$, local flow acceleration over the suction side and back eddies on the pressure side of Model 1 can be seen in Figure 10a. For $\theta =25^{\circ }$ and $50^{\circ }$, the Kelvin-Helmholtz instability-driven roll-up of the separated shear layer culminates in the formation of coherent vortex structures at the leading and trailing edges, ultimately leads to the formation of a turbulent wake dominated by an alternating Von Kármán vortex street. In the close-up perspectives, the wake vortex street downstream of the arched and cambered deflector exhibits a deflected configuration due to asymmetric force distribution between its upper and lower surfaces, resulting in shed vortices with distinct intensity and dimensions across different flow boundaries. It is further justified in Figure 10b,c. In comparison, symmetric airfoils, such as NACA0012, demonstrate comparable flow separation characteristics on both surfaces under post-stall conditions, thereby generating relatively regular vortex streets characterized by quasi-periodic counter-rotating vortex pairs [45].

**Figure 11:**
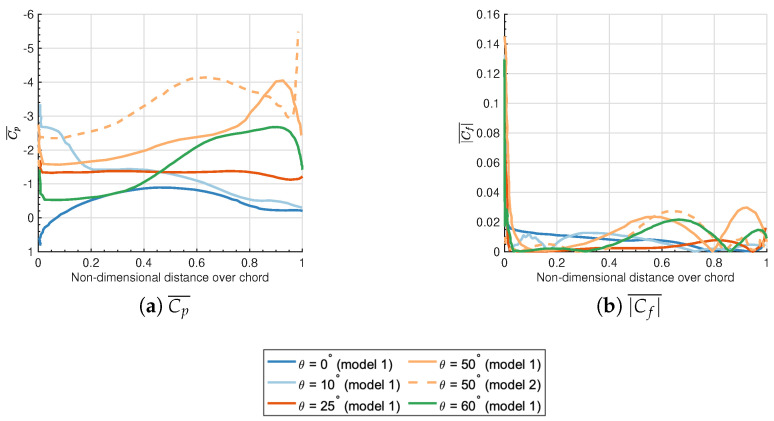
Distributions of mean pressure coefficients $\bar{C_{p}}$ and modulus of skin-friction coefficients $\bar{|C_{f}|}$ over the suction side of the deflector.

**Figure 12:**
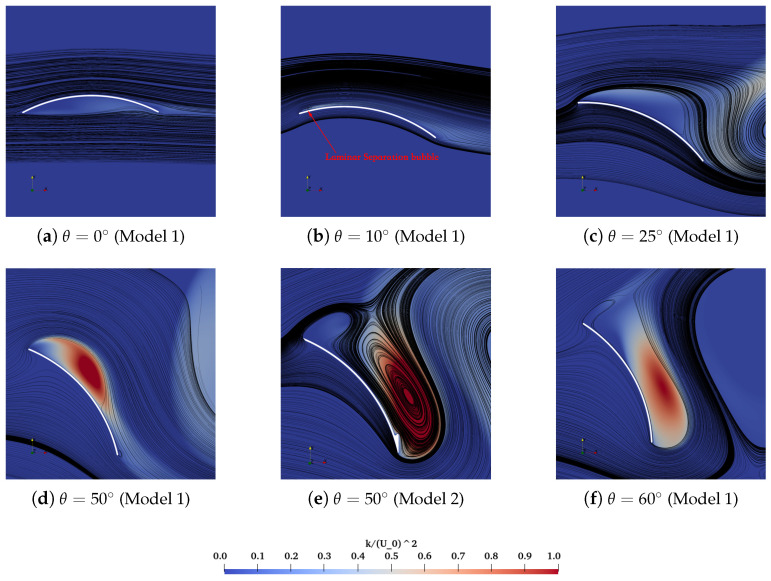
Instantaneous non-dimensional turbulent kinetic energy $k/{U_{0}}^{2}$ and the streamlines around the deflector with the variation of inclination angles.

Based on the interrelations among force coefficients, $C_{p}$, $C_{f}$ in addition to turbulence intensities, the underlying mechanism of loads on and flow separation over deflectors has been clearly discussed here. Bak et al. [46] has defined at least two distinct stall levels with θ as the “double stall” feature of aerofoil flows, which confirms the relations with the formation and breaking of LSB, i.e., the flow separation or reattachment behaviors. The comparable observation of the non-linear variation of $C_{L}$ with θ is also confirmed in the experiment [47]. In this section, the plateau of $C_{L}$ for $\theta =10^{\circ }$–$35^{\circ }$ is discussed with the local distributions of $C_{p}$ and $C_{f}$ over the suction side of the deflector in Figure 11, accompanied by the instantaneous non-dimensional turbulent kinetic energy $k/{U_{0}}^{2}$ and the streamlines around the isolated deflector in Figure 12. For $\theta =0^{\circ }$, the streamlines remain attached at the majority of the suction side (Figure 12a), except for the eventual separation close to the trailing edge due to the critical adverse pressure gradient. The flattening region of $C_{p}$ after the non-dimensional distance 0.8 over the chord, as well as the zero-crossing of $C_{f}$, also verify the occurrence of separation quantitatively. For $\theta =10^{\circ }$, the small-form LSB is generated at the leading edge (Figure 12b), which is attributed to the separation of laminar flow and the reattachment after the transition of separated shear layers to turbulence. The similar LSB formation close to the leading edge of the NACA0012 airfoil is also addressed in the PIV measurements [48]. It drives the continuous rising of $C_{L}$ at smaller θ. With traveling from the leading edge to the trailing edge, $C_{p}$ exhibits a steep gradient transition followed by a plateau region near the leading edge, subsequently reverting to an adverse pressure gradient distribution. This flow characteristic, when combined with the dual zero-crossing positions observed in the $C_{f}$ profile, provides quantitative evidence corroborating the existence of an LSB. As depicted in Figure 12c, the original small-form separation bubble elongates to the trailing edge considerably until the moment that it fails to reattach to the surface with a comparatively lower *k* level at $\theta =25^{\circ }$. This phenomenon is specified as a “short bubble burst” with the flat formation of $C_{p}$ and $C_{f}$ (Figure 11), contributing to the declination or plateau of $C_{L}$ for $\theta =10^{\circ }$–$35^{\circ }$. The initial stall has occurred. Then, it is reported that the breaking LSB turns into a long-form separation bubble with the larger θ when the reattachment occurs as the transition of laminar-state shear layers into turbulence states [2,49,50]. Within this hydrodynamic context, the continuously attached long-form separation bubble, with higher turbulence intensities (Figure 12d), gives rise to the increased lift for $\theta =35^{\circ }$–$50^{\circ }$ in Figure 9. Figure 12f has proved that the rising of $C_{L}$ is, however, unsustainable owing to the critical flow separations at the suction side, not to mention the intricate distributions of $C_{p}$ and $C_{f}$. This corresponds to the final stall that cannot be recovered quickly.

The primary distinction between the two models is the enhanced performance of $C_{L}$ in Model 2 at $\theta =40^{\circ }$–$60^{\circ }$, which is caused by a quicker transition to turbulence and the reattachment of the shear layer. Here, a particular emphasis is placed on the flow fields of two models at $\theta =50^{\circ }$ as a case for comparison. Compared to Model 1, Model 2 exhibits a stronger negative pressure field in Figure 11a, resulting in a greater lifting force. Additionally, the effectiveness of closing the separation bubble to maximize lift generation is supported by the distributions of $C_{f}$ and the instantaneous streamlines. Corresponding to the reattachment and the eventual separation, the first and second zero points of $C_{f}$ of Model 2 at $\theta =50^{\circ }$ occur at 0.28 and 0.85, respectively. Furthermore, the separation bubble depicted in Figure 12e is significantly larger than the one shown in Figure 12d.

Ultimately, the isolated case with the $\theta =10^{\circ }$ prevails as the decent performances of $C_{L}$ and γ globally. Considering the better lift performances and efficiencies of Model 1 against Model 2 for $\theta =10^{\circ }$, it is suggested to use Model 1 as the leader deflector for further studies on tandem deflectors.

### 3.3. Phase 2: The Metamodel of Force Coefficients of Tandem Deflectors

**Figure 13:**
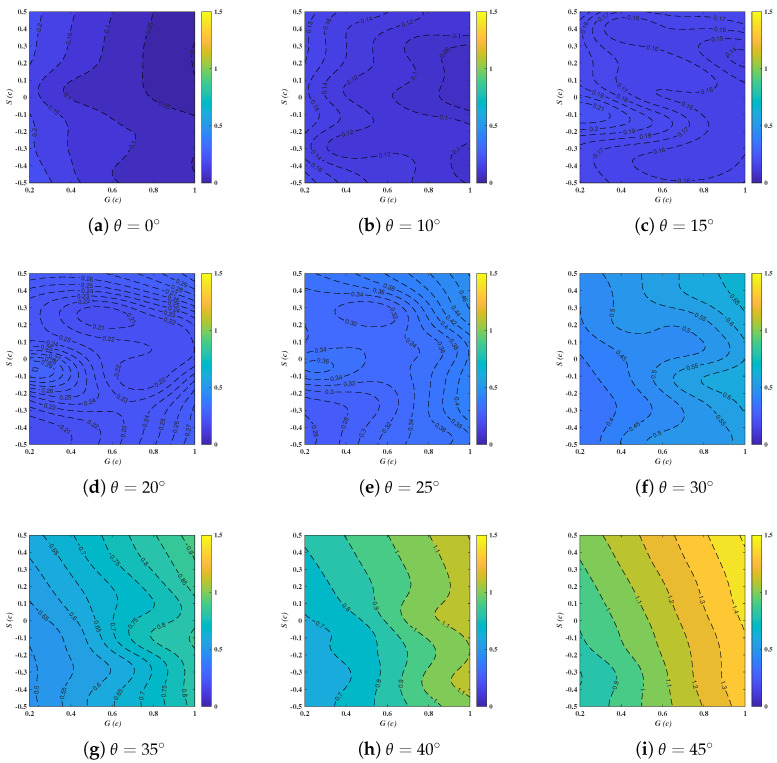
Response surfaces of $C_{d}$ with respect to *G*, *S* and θ.

The metamodels of $C_{D}$ and $C_{L}$ are trained following the procedure in Figure 7, and the correlations between the force coefficients and the independent variables are demonstrated as follows. It exposes that the inclination angles of the follower deflector exhibit a near-linear influence on $C_{D}$, from Figure 13. Interestingly, when S∼0.5c and G∼1.0c, the global maximum of $C_{D}$ occurs at $\theta =45^{\circ }$, whereas the minimum appears at $0^{\circ }$. This qualitatively suggests that θ exerts a more pronounced influence on $C_{D}$ than the other two parameters. Moreover, the impacts of *G* and *S* on $C_{D}$ are considerably non-linear and intricate, especially for $\theta =15^{\circ }$–$30^{\circ }$. Various peaks and valleys of $C_{D}$ are shown in Figure 13c–f, in addition to the extensive and stochastic distributions of contour lines. The flow interactions for the in-line and staggered layouts in the tandem configuration might explain the pattern [15]. For instance, the jet flow generated by the fore deflector can directly impinge on the suction side of the aft one, suppressing the flow separation from the boundary-layer region. It also hints at the impracticality of regression analyses or curve fittings to address the non-linear and high-order dependencies among output and input variables, demonstrating the superb quality of metamodelings.

For $\theta =0^{\circ }$–$10^{\circ }$, the lowest $C_{D}$ shows for S>0 with G∼1.0c synchronously, while the peaks in $C_{D}$ are found when *G* is around 0.2c. The influence of *S* is less significant compared to that of *G*. The latter shows an inverse correlation with $C_{D}$, suggesting that reduced vertical spacing increases the resistance of the entire tandem system at small inclinations. For $\theta =35^{\circ }$–$45^{\circ }$, it seems that *G* presents a strong positive association with $C_{D}$, exhibiting a stark contrast to the result for $\theta =0^{\circ }$–$10^{\circ }$. The synergistic effect of *G* and *S* on $C_{D}$ gradually diminishes with increasing θ, to the extent that at $45^{\circ }$, the influence of *S* on $C_{D}$ is significantly less pronounced than that of *G*.

**Figure 14:**
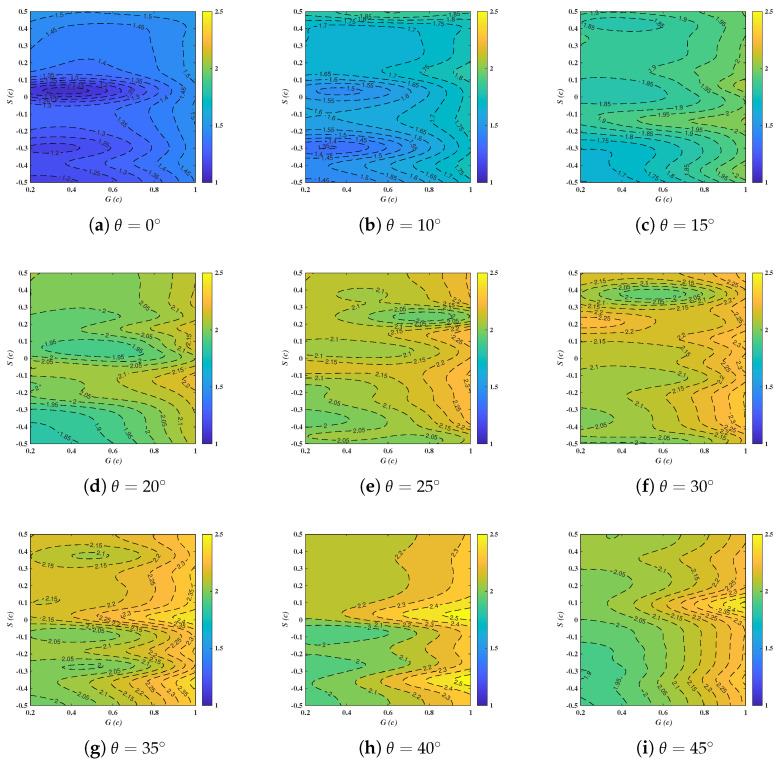
Response surfaces of $C_{l}$ with respect to *G*, *S* and θ.

Figure 14 captures the distribution of $C_{L}$ with *G*, *S* and θ. The non-linear dependences of $C_{L}$ on the three variables are detected by the irregularly shaped contour lines, which is in accordance with the findings of $C_{D}$ at $\theta =15^{\circ }$–$30^{\circ }$ but occurs for a wider range of θ here. In comparison with $C_{D}$, the heightened non-linearity characteristics among $C_{L}$ and input variables are manifested. This is triggered by flow separation or attachment over the suction side of the deflector, causing the oscillation of negative pressure fields [2]. The apparent positive associations between $C_{L}$ and θ are merely identified for $\theta =0^{\circ }$–$15^{\circ }$. The variations of $C_{L}$ are within 1.85–2.50 at θ exceeding $20^{\circ }$, indicating the stronger synergistic effects of *G* and *S* are not trivial at larger θ.

In detail, it can be seen that $C_{L}$ increases progressively with the rising of *G*, especially for S=−0.5c–0. This tendency is, however, not of significance for S>0. In agreement with the experimental and theoretical analyses [14], the influence of *S* is not quite considerable compared to *G*, particularly at $\theta =45^{\circ }$. It is reported that the maximum available lift forces for tandem wings decreases with increasing *S* and occurs for varying θ and *G* within a certain limit [14], which can be further confirmed in the present study (Figure 14). The *G* factor critically governs the aerodynamic interaction between tandem deflectors by determining the degree to which the rear deflector is immersed in the downwash flow generated by the forward one [11]. For instance, under conditions of reduced *G* combined with substantial *S*, the rear deflector becomes fully encapsulated within the wake region of the forward deflector. Furthermore, the *G* dimension plays a critical role in governing both the pressure differential gradient and the intensity of flow interactions between the deflectors [14,29].

It is of great interest to note that the maximum and the minimum $C_{L}$ are both located at S∼0, corresponding to the zero-stagger scenarios. On the one hand, the decline of *G* simultaneously means the proximity of the trailing edge of the fore deflector with the leading edge of the aft one. This leads to the underdeveloped leading-edge vortex prematurely impacting the trailing deflector, which restricts the downstream surface to a low dynamic pressure wake region, ultimately resulting in the minima of $C_{L}$ [11]. On the other hand, the peaks in $C_{L}$ can be observed in parallel with G∼1.0c and $\theta =35^{\circ }$–$45^{\circ }$, hinting that the expansion of *G* can overcome the issues associated with zero-stagger scenarios. As previously demonstrated results of $C_{D}$, the peaks also emerged at $\theta =35^{\circ }$–$45^{\circ }$. However, based on the lift performance and efficiency analysis, it is important to note that the optimized case cannot be directly concluded from the qualitative illustrations provided here.

The significance of each input variable is quantitatively assessed using the Random Forest (RF) method, which effectively analyses non-linear relationships and complex interactions among variables [51]. The importance of a variable in RF is commonly determined using the out-of-bag (OOB) Permuted Predictor Delta Error property, which is part of the TreeBagger class in MATLAB R2023b. Detailed calculations can be found in Janitza et al. [52]. Here in Table 4, the relative importance index for each independent variable quantifies the fractional contribution of each component to the overall significance, expressed as the proportional weight (percentage) of individual portions relative to the total system. The dominance of θ in governing the variations of $C_{D}$ and $C_{L}$ is justified with the relative importance indices 67.83% and 49.50%, respectively. *G* ranks the second position then followed by *S*, aligning with the earlier discussions. The values of 49.50% for $C_{L}$ compared to 67.83% for $C_{D}$ indicate that the impact of θ on $C_{L}$ diminishes when it exceeds $20^{\circ }$. Furthermore, the higher importance indices of *G* and *S* in $C_{L}$ relative to $C_{D}$ underscore the greater significance of spatial configurations, which may be closely connected to pressure distributions.

### 3.4. Phase 3: Hydrodynamics Implications of the Optimized Tandem Deflectors

In this section, we present an optimized tandem deflector case obtained through the metamodeling workflow introduced in Section 2.3. The optimization aims to maximize $C_{L}$ and γ. The candidate case of tandem deflectors selected using the ultimate MOGA from the metamodeling workflow is shown in Table 5, which includes comparisons between predicted data and CFD results. The effectiveness and accuracy of the metamodel have been validated, showing less than 1% relative error. The optimized values for *G* and *S* indicate that a vertical spacing equal to one chord length and a nearly zero stagger satisfy the established criteria. This aligns with the analysis of the response surfaces of $C_{L}$ in Figure 14. Provided the opposing relationship between drag and lift components as noted by Wang et al. [26], the final optimized configuration features $\theta =19.272^{\circ }$, demonstrating commendable lift performance while maintaining low resistance.

To obtain better knowledge about the improved properties of the tandem deflectors, the isolated cases of Model 1 with $\theta =10^{\circ }$ and $20^{\circ }$ are utilized for further comparison. $C_{L}$ of the optimized tandem case is 2.194 versus 1.882 and 1.573 for the isolated case with $\theta =10^{\circ }$ and $20^{\circ }$, with the improvements of 16.6% and 39.5%, respectively. Nevertheless, the improvements of γ of the optimized tandem case are not as remarkable as $C_{L}$. The γ=11.309 against 18.031 and 3.547 for the isolated case with $\theta =10^{\circ }$ and $20^{\circ }$, respectively. The γ of the optimized tandem case lags behind the isolated case with $\theta =10^{\circ }$ but is still larger than 10, which is acceptable according to the aquacultural engineering practices. As stated before, it is rather challenging to locate the solutions that can drastically meet Pareto’s optimal front, particularly for these devices optimized for cost minimization and efficiency enhancement.

**Figure 15:**
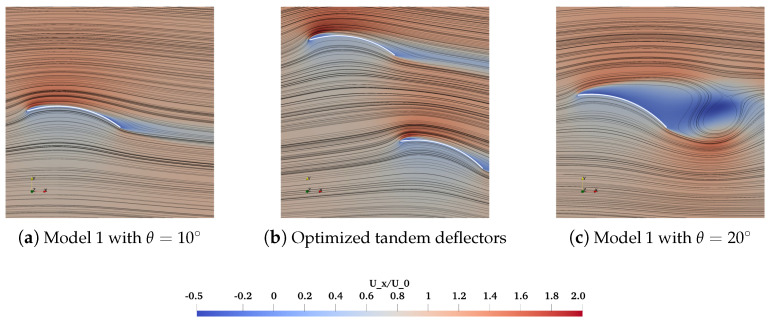
Instantaneous non-dimensional streamwise velocity $U_{x}/U_{0}$ and the streamlines around the isolated and tandem deflectors.

The enhancements observed in the optimized tandem configurations are showcased through the distributions of transient velocity fields and streamlines, as illustrated in Figure 15. Similarly, an LSB forms on the upper surface of the fore deflector, as proved in Figure 15a,b. The evolution of streamlines around the fore deflector resembles that of Model 1 with $\theta =10^{\circ }$. To underline the superiority of the tandem configuration in flow interactions, the Model 1 case with $\theta =20^{\circ }$ (Figure 15c) is especially compared with the optimized tandem case. One can note the significant flow separation at the upper surface of Model 1 with $\theta =20^{\circ }$. It cannot be replicated for the follower deflector (Figure 15b), which is suppressed by the downwash flow from the fore deflector. A comparable LSB is instead formed at the follower deflector and remains attached. It implies that the onset of the initial stall has been delayed at $\theta =19.272^{\circ }$, arising from the flow interaction effects and confirmed in Scharpf and Mueller [11] experimentally.

## 4. Conclusions and Outlooks

The flow over the cambered deflectors at $Re=1\times 10^{5}$ is investigated using the workflow combining Kriging metamodeling with k−ω SST URANS simulations. The accuracy of the numerical method is validated using the force coefficients of a cambered trawl door model. The hydrodynamics of the isolated cambered deflectors, both with or without bio-inspired features, are modeled initially using CFD simulations. In this context, the optimized isolated case is used to create the tandem-configured deflectors. Herein, the dependences of the crucial parameters, including *G*, *S*, as well as θ, to force coefficients and flow distributions are discussed qualitatively and quantitatively. The conclusions are drawn as follows:

(1) For the isolated deflector, the “double stall” phenomenon of $C_{L}$ over θ has been confirmed. This phenomenon is linked to the formation, movement, and breaking of LSB at the upper surface of the deflector. The flow separations and reattachments have been effectively captured using $C_{p}$ and $C_{f}$.

(2) The bio-inspired features significantly enhance lift forces compared to non-bionic designs for $\theta =30^{\circ }$–$60^{\circ }$, as the larger LSB can be continuously attached at higher inclination angles. The non-bionic isolated deflector with the $\theta =10^{\circ }$ prevails as the decent performances of $C_{L}$ and γ globally, which is chosen for the following studies of tandem deflectors.

(3) Based on the trained metamodel of $C_{D}$ and $C_{L}$ with respect to *G*, *S* and θ, it suggests that θ plays a more vital role in $C_{D}$ compared to the other two parameters, while the impacts of *G* and *S* on $C_{D}$ are considerably non-linear and intricate, especially for $\theta =15^{\circ }$–$30^{\circ }$. By the lights of the RF method describing the importance of input variables, it can further confirm that *G* has a greater influence than *S*.

(4) In comparison with $C_{D}$, the heightened non-linearity characteristics among $C_{L}$ and input variables are manifested. The apparent positive associations between $C_{L}$ and θ are merely identified for $\theta =0^{\circ }$–$15^{\circ }$, $C_{L}$ keeps 1.85–2.50 afterwards. The influence of *S* is not quite considerable compared to *G*, which can further be substantiated in the importance ranking.

(5) θ essentially sets the baseline lift, drag, and stall propensity, while *G* and *S* determine the intensity and nature of the inter-deflector flow coupling, together shaping the overall hydrodynamic performance.

(6) Lastly, an optimized tandem case is obtained from the ultimate MOGA step, which is desirable for cost minimizations and lift improvements. The superiorities of hydrodynamics are also emphasized via the comparisons against the isolated deflector cases.

It should be noted that the present two-dimensional modeling offers much lower computational cost while still capturing the majority of pertinent flow characteristics. The flow behaviors across the spanwise direction of deflectors are, however, overlooked. Therefore, it is anticipated to use three-dimensional large eddy simulations (LES) to capture more flow details between tandem deflectors in future studies.

## Figures and Tables

**Table 1:** Variation ranges for the investigated parameters.

Parameters	*G*	*S*	θ
Range	0.2*c*–1.0*c*	−0.5*c*–0.5*c*	$5^{\circ }$–$45^{\circ }$

**Table 2:** Grid independence examination using the validated case with inclination angle $30^{\circ }$. RD refers to the relative difference between the numerical and experimental results.

Cases	Outer Grid	Near-Field	Grid	$C_{D}$	$C_{L}$	RD ($C_{D}$/$C_{L}$) [%]
	Size [m]	Size [m]	Number			
M1	0.030	9.38 × 10^−4^	4.05 × 10^6^	0.583	1.512	−2.85%/12.54%
M2	0.025	7.81 × 10^−4^	6.82 × 10^6^	0.602	1.410	0.31%/4.92%
M3	0.020	6.25 × 10^−4^	1.29 × 10^7^	0.588	1.350	−2.07%/0.45%
M4	0.015	4.69 × 10^−4^	2.38 × 10^7^	0.595	1.341	−0.77%/−0.15%
Exp. [43]	-	-	-	0.600	1.343	-

**Table 3:** Comparisons of the simulated $C_{D}$ and $C_{L}$ against the experimental and simulated results in Li et al. [43]. See Table 2 for the definition of RD.

θ	$C_{D}$/$C_{L}$	$C_{D}$/$C_{L}$	RD	$C_{D}$/$C_{L}$	RD
	(Exp. [43])	(CFD [43])		Present Study	
$10^{\circ }$	0.234/1.000	0.224/1.064	−4.35%/6.44%	0.233/0.961	−0.26%/−3.95%
$20^{\circ }$	0.427/1.417	0.410/1.505	−3.97%/6.22%	0.434/1.366	1.49%/−3.60%
$30^{\circ }$	0.600/1.343	0.600/1.508	0.00%/12.29%	0.588/1.350	−2.07%/0.45%
$40^{\circ }$	0.840/0.993	0.820/1.137	−2.38%/14.43%	0.797/1.012	−5.17%/1.91%

**Table 4:** Relative importance index for each input variable.

	*G*	θ	*S*
$C_{D}$	23.56%	67.83%	8.62%
$C_{L}$	30.59%	49.50%	19.91%

**Table 5:** Candidate case of tandem deflectors through the metamodeling workflow.

	G(c)	θ [Degree]	S(c)	$C_{D}$	$C_{L}$	γ
Metamodel	0.997	19.272	0.068	0.193	2.174	11.264
CFD				0.194	2.194	11.309
|Relative error|	-	-	-	0.72%	0.90%	0.40%

## Data Availability

Data will be made available on request.

## Abbreviations

The following abbreviations are used in this manuscript:

$U_{0}$	Freestream velocity
Re	Reynolds number
*G*	Gap
*S*	Stagger
θ	Angle of attack
r/c	Relative camber
$C_{D}$	Mean drag coefficient
$C_{L}$	Mean lift coefficient
γ	Lift-drag ratio
$C_{f}$	Skin-friction coefficient
$C_{p}$	Pressure coefficient
*k*	Turbulent kinetic energy
ω	Turbulent specific dissipation rate
ϵ	Turbulent dissipation rate
CFD	Computational Fluid Dynamics
EARSM	Explicit Algebraic Reynolds Stress Model
HLTD	Hyper-Lift Trawl Door
LSB	Laminar Separation Bubbles
MOGA	Multi-Objective Genetic Algorithm
PIV	Particle Image Velocimetry
RD	Relative Divergences
RF	Random Forest
SST	Shear Stress Transport
URANS	Unsteady Reynolds-Averaged Navier–Stokes
